# Targeted Lipidomics for Characterization of PUFAs and Eicosanoids in Extracellular Vesicles

**DOI:** 10.3390/nu14071319

**Published:** 2022-03-22

**Authors:** Madlen Reinicke, Saikal Shamkeeva, Max Hell, Berend Isermann, Uta Ceglarek, Mitja L. Heinemann

**Affiliations:** Institute of Laboratory Medicine, Clinical Chemistry and Molecular Diagnostics, Leipzig University Hospital, Paul-List-Str. 13-15, 04103 Leipzig, Germany; madlen.reinicke@medizin.uni-leipzig.de (M.R.); saikal.shamkeeva@medizin.uni-leipzig.de (S.S.); mhell97@gmx.de (M.H.); berend.isermann@medizin.uni-leipzig.de (B.I.); uta.ceglarek@medizin.uni-leipzig.de (U.C.)

**Keywords:** extracellular vesicles, quantitative lipidomics, eicosanoids, pre-analytics

## Abstract

Lipids are increasingly recognized as bioactive mediators of extracellular vesicle (EV) functions. However, while EV proteins and nucleic acids are well described, EV lipids are insufficiently understood due to lack of adequate quantitative methods. We adapted an established targeted and quantitative mass spectrometry (LC-MS/MS) method originally developed for analysis of 94 eicosanoids and seven polyunsaturated fatty acids (PUFA) in human plasma. Additionally, the influence of freeze–thaw (FT) cycles, injection volume, and extraction solvent were investigated. The modified protocol was applied to lipidomic analysis of differently polarized macrophage-derived EVs. We successfully quantified three PUFAs and eight eicosanoids within EVs. Lipid extraction showed reproducible PUFA and eicosanoid patterns. We found a particularly high impact of FT cycles on EV lipid profiles, with significant reductions of up to 70%. Thus, repeated FT will markedly influence analytical results and may alter EV functions, emphasizing the importance of a standardized sample pretreatment protocol for the analysis of bioactive lipids in EVs. EV lipid profiles differed largely depending on the polarization of the originating macrophages. Particularly, we observed major changes in the arachidonic acid pathway. We emphasize the importance of a standardized sample pretreatment protocol for the analysis of bioactive lipids in EVs.

## 1. Introduction

### 1.1. Extracellular Vesicles

Extracellular Vesicles (EVs) are a heterogeneous group of phospholipid bilayer-enclosed particles that are naturally secreted into the extracellular matrix by many different cell types [1]. Their role in intercellular cargo transfer has led to a high interest in EVs, with the aims of identifying new biomarkers and therapeutic targets and further elucidating EV functions [2]. EVs may contain various type of biological materials, such as nucleic acids (mRNA, miRNA, DNA), lipids, and proteins. Depending on their respective origin EVs may induce different responses within target cells, such as cell migration, apoptosis, and cytokine release [3,4]. Among others, EVs are known contributors to inflammatory responses as well as to inflammation-resolving effects [3,5,6].

The composition and function of EV miRNAs and EV proteins have been the subject of extensive investigations, as have EV biomarker models [7,8]. Recently, there has been a rise of interest into the lipid composition of EV membranes [7,9], as these contain various lipid species including bioactive lipids such as polyunsaturated fatty acids (PUFAs) and eicosanoids. However, very little is known about the composition and function of these bioactive lipids in EVs, reflecting at least in part a lack of adequate analytical tools [7].

### 1.2. Bioactive Lipids: Polyunsaturated Fatty Acids and Their Metabolites

PUFAs serve as structural membrane components and are involved in signalling, functioning as regulatory molecules [10]. PUFAs can be classified into two major groups; ω-6 PUFA-derived metabolites originate from arachidonic acid (ARA) and linoleic acid (LA) and have generally pro-inflammatory functions, whereas ω-3 PUFAs originate from alpha linolenic acid (ALA), eicosapentaenoic acid (EPA), and docosahexaenoic acid (DHA), convey anti-inflammatory signals, and contribute to the resolution of inflammation [10,11] (Supplemental Figure S1). ARA-derived eicosanoids are particularly well understood and are known to be involved in a wide range of regulatory processes, such as cytokine production, cell differentiation, proliferation, and migration, neurological development, inflammation, and antigen presentation [12].

Release and metabolism of membrane-bound ARA is tightly regulated, leaving little ARA as a substrate for eicosanoid biosynthesis. Activation of phospholipases induces release and accumulation of ARA, enhancing its enzymatic oxidation by cyclooxygenases (COX), lipoxygenases (LOX), and cytochrome P450 (CYP) enzymes [13]. The eicosanoid products of these enzymes differ. Prostaglandins or thromboxanes are synthesized mainly by COX-2, while 8-hydroxyeicosatetraenoic acid (8-HETE) and 12-hydroxyeicosatetraenoic acid (12-HETE) are products of 8- and 12-LOX, respectively. Other HETEs can be produced by CYPs as well.

In complex biological samples, bioactive lipids are present freely or bound to proteins such as albumin, and can be embedded in EV and cell membranes as phospholipid esters [11]. However, the composition and function of EV bioactive lipids is not fully understood.

### 1.3. Analysis of Bioactive Fatty Acid Metabolites in EVs

During inflammatory processes, PUFAs and their metabolites can be synthetized by, among other things, cells of the innate immune system such as macrophages or mast cells, then released as free form, protein bound, or EV forms [4,14]; these lipids can then later be released from EV membranes [7,11,12]. Because EVs may contain many of the enzymes involved in PUFA and eicosanoid metabolism, such as COX or LOX, EVs can modify bioactive lipid metabolism in plasma or target cells [9,11,15,16,17,18].

In order to understand the lipid composition of EVs, an increasing number of different EV isolation strategies as well as multiple lipid extraction protocols and mass spectrometry methods for EV analysis have been proposed (see Supplemental Table S1). While there are current lipidomic methods that represent untargeted approaches, other methodologies are able to quantify lipids by using targeted approaches, most commonly liquid chromatography–tandem mass spectrometry (LC-MS/MS). However, none of the currently established methods for analysing EV lipids is able to quantify large numbers of fatty acids and their metabolites. To analyse PUFAs and eicosanoids from EVs, pre-analytical influencing factors have to be taken into account: First, the choice of EV isolation methodology already impacts the lipidomic results, as isolation time and physical and chemical stress can alter the lipid composition of the EV preparation. Particularly time-consuming protocols such as ultracentrifugation (UC) facilitate eicosanoid oxidation and enzymatic activity during isolation [19,20]. These pre-analytic alterations are defined by temperature, additives, and duration of the EV isolation [21,22] and are a known issue in eicosanoid analysis; they can have a severe impact on analyte concentrations. Additionally, precipitation methods can lead to co-isolation of protein-bound eicosanoids and lipid-modifying enzymes such as cyclooxygenases [23]. In contrast, filtration-based methods such as tangential flow filtration (TFF) can overcome these issues thanks to their shorter isolation times and depletion of free eicosanoids and smaller proteins. After isolation, the amount of freeze–thaw (FT) cycles can impact the concentration and composition of EV preparations [24].

Eicosanoid analysis is impacted by lipid extraction in organic solvents, most commonly achieved using chloroform/methanol [15,16,17,18,25,26,27,28,29]. These methods represent well-established extraction protocols for highly abundant mainly non-polar lipids. However, chloroform/methanol extraction protocols are not applicable to simultaneous PUFA and eicosanoid analysis due to their differing polarities and abundances. Alternative extraction protocols have been developed; however, these have not been used for EV lipid analysis to date [21,22].

The aim of our study was the development of a reliable sample preparation protocol for EV lipids using quantitative targeted LC-MS/MS on seven PUFAs and 94 eicosanoids in EVs. The novel protocol was applied to the quantitative comparative analysis of bioactive lipids in EVs derived from M1 and M2 macrophages.

## 2. Materials and Methods

### 2.1. EV Isolation

#### 2.1.1. Cell Culture

The human monocytic leukemia cell line THP-1 (American Type Culture Collection, Rockville, MD, USA) was cultured as previously described [30,31,32,33] in Very Low Endotoxin—Roswell Park Memorial Institute 1640 (VLE-RPMI1640) liquid medium (Biochrom Gmbh, Germany) supplemented with stable glutamine (2.0 g/L NaHCO_3_), 10% Fetal Bovine Serum (FBS Superior, Biochrom Gmbh, Berlin, Germany), and 500 units penicillin, 500 µg streptomycin, and 1.25 µg Amphotericin B (Gibco, Grand Island, NE, USA). Cells were incubated in a humidified incubator at 37 °C with 5% CO_2_. THP-1 cells were seeded at 0.2 × 10^6^ per mL in T175 flasks and treated with 25 nM phorbol 12-myristate 13-acetate (PMA, Sigma Aldrich, Hamburg, Germany) for 48 h in order to differentiate into a macrophage-like state (M0) [30]. Differentiated cells were washed once with EV-depleted medium.

M0 macrophages were then treated with EV-depleted media supplemented with either 20 ng/mL interferon-γ (IFN-γ) and 1 µg/mL lipopolysaccharide (LPS) for 24 h in order to polarize them into an M1-like state, or with 20 ng/mL Interleukin 4 (IL-4) for 24 h in order to polarize them into an M2-like state [30,32]. Cell culture supernatants were subsequently harvested for EV isolation.

#### 2.1.2. Reverse-Transcription Quantitative Polymerase Chain Reaction (RT-qPCR)

Gene expression of the differently polarized macrophages was determined by RT-qPCR. Total RNA was isolated using TRIzol RNA isolation reagent (ThermoScientific, Berlin, Germany) following the manufacturer’s protocol. cDNA was generated using RevertAid First Strand cDNA Synthesis Kit (Thermo Scientific, Berlin, Germany). Quantitative RT-qPCR was accomplished using Takyon™ No ROX SYBR 2X MasterMix blue dTTP (EuroGentec, Cologne, Germany) in 384-well plates. RT-qPCR was performed targeting M1/M2 marker expression (the full list of primers can be found in Supplementary Table S2). Gene expression was determined relative to beta-actin expression and to resting M0 macrophages.

#### 2.1.3. Generation of EV-Depleted Media

EV-depleted media for THP-1 cell culture were generated as previously described [34,35]. Briefly, complete cell culture media VLE-RPMI 1640 liquid medium (Biochrom Gmbh, Berlin, Germany) supplemented with stable glutamine (with 2.0 g/L NaHCO_3_), 10% fetal bovine serum (FBS Superior, Biochrom Gmbh, Berlin, Germany) and 500 units penicillin, 500 µg streptomycin, and 1.25 µg Amphotericin B (Gibco, Grand Island, NE, USA) was filtered through a tangential flow filtration (TFF) system, equipped with a 500 kDa Molecular Weight Cut Off (MWCO) hollow fiber filtration module (Repligen, Waltham, MA, USA) while constantly maintaining a transmembrane pressure of <1.5 PSI. Filtrate was considered EV-free and used for cell culture.

#### 2.1.4. EV Isolation and Purification

EVs were purified as previously described [34,35]. Briefly, 120 mL cell culture supernatants of M0, M1, and M2 THP-1 cell cultures were passed through a modified polyethersulfone (mPES) filter with a 100 nm pore size (Merck, Darmstadt, Germany) in order to eliminate floating cells, dead cells, and cell debris from the solution. The filter was then rinsed with 50 mL of 1X Dulbecco’s phosphate-buffered saline (DPBS). In a second step, the sample was concentrated and purified by TFF. Briefly, the filtrate from step one was transferred to a TFF system (Repligen, Waltham, MA, USA) equipped with a 500 kDa molecular weight cutoff (MWCO) hollow fiber filtration module. The sample was diafiltrated six times with 1X DPBS and then concentrated to a final volume of 10 mL. Transmembrane pressure was constantly monitored via pressure transducers and did not exceed 1.5 PSI.

### 2.2. EV Characterization

#### 2.2.1. Nanoparticle Tracking Analysis (NTA)

The size and particle concentrations of the purified EVs were assessed by Brownian microscopy using a NanoSight LM14 analyzer (Malvern Panalytical, Malvern, UK). Briefly, the samples were prediluted with PBS to a total volume of 500 µL and infused into a cuvette (d = 500 µm) at a temperature of 22 °C. Illumination with a laser beam (λ = 532 nm) caused light scattering of particles present in the solution. Scattered light was collected with a 20× microscope lens and captured by a CMOS camera. A total of three videos (length of 30 s each, 25 frames per second) were recorded and processed by Nanotracking Analysis Software, with the analysis based on tracking individual particles in the acquired videos. The obtained mean square displacement as well as the solvent viscosity (phosphate-buffered saline (PBS): 0.93 mPa s) enabled particle diameter calculation.

#### 2.2.2. Pierce Micro BCA Protein Assay

The protein contents of the isolated EVs were measured using a Pierce Micro-bicinchoninic acid (BCA) Protein Assay (Thermo Scientific, Berlin, Germany), according to the manufacturer’s instructions. Absorbance was measured at 562 nm using a Multiscan FC Microplate Photometer (Thermo Scientific, Berlin, Germany).

#### 2.2.3. Protein Profiling by Flow Cytometry

Commonly-described EV marker proteins were profiled using the MACSPlex Exosome kit (Miltenyi Biotec, Bergisch Gladbach, Germany), according to the manufacturer’s instructions.

### 2.3. Quantitative LC-MS/MS Analyses

#### 2.3.1. PUFAs

Targeted LC-MS/MS analysis for quantification of the seven PUFAs was performed according to our previously published method [36], with slight modifications. A Shimadzu LC system (Duisburg, Germany) configured with an HTS PAL autosampler from CTC Analytics (Zwingen, Switzerland) and including online solid-phase extraction (SPE) was coupled to a SCIEX QTRAP^®^ 5500 mass spectrometer (Framingham, MA, USA) equipped with a Turbo V™ ion spray source operating in negative ion mode. Modifications of the gradient elution were implemented for method separation and analysing only PUFAs (see Supplemental Figure S2A). The adapted gradient was as follows: 0 to 90% eluent B in 8 min (eluent A: H_2_O/acetonitril/formic acid 63:37:0.02 *v*/*v*/*v*, eluent B: iPrOH/acetonitril 50:50 *v*/*v*), 90% B for 2 min, re-equilibration of the columns for 2 min, flow rate of 0.6 mL/min.

#### 2.3.2. Eicosanoids

Quantitative LC-MS/MS analysis of 94 eicosanoids, as published previously by our group [36], was optimized and transferred to a more sensitive mass spectrometer. An LC system from Shimadzu (Duisburg, Germany) equipped with a PAL HTC-xt autosampler from CTC Analytics (Zwingen, Switzerland) and including online-SPE was coupled to a SCIEX QTRAP^®^ 6500+ mass spectrometer (Framingham, MA, USA) equipped with a Turbo V™ ion spray source operating in negative ion mode. Chromatographic separation (see Supplemental Figure S2B) was achieved on a core-shell LC column (Kinetex C18, 100 × 2.1 mm i.d., 2.6 µm particle size, Phenomenex, Aschaffenburg, Germany) kept at 50 °C. The gradient elution was as follows: 15 to 70% eluent B in 13 min (eluent A: H_2_O/MeOH/formic acid 95:5:0.05 *v*/*v*/*v*, eluent B: MeOH/H_2_O/formic acid 95:5:0.05 *v*/*v*/*v*), 100% B for 2 min, re-equilibration of the columns for 2 min, flow rate of 0.7 mL/min.

#### 2.3.3. Optimization of Sample Preparation for EV Analysis

Experiments were conducted using pooled EV isolations derived from resting (M0) and M1 or M2 polarized macrophages. Lipid extraction was combined with protein precipitation using MeOH*BHT/H_2_O*ZnSO_4_ (80:20 *v*/*v*), final concentration 17.8 g/L ZnSO_4_, 56 mg/L butylated hydroxytoluene (BHT) (37). In comparison, alternative approaches using Methanol (containing 56 mg/L BHT), n-hexane/iPrOH (60:40 *v*/*v*), and acetonitrile/H_2_O (80:20 *v*/*v*) were tested. Results were expressed as the ratio of analyte peak area to internal standard (IS) peak area. For statistical analysis, a one-way analysis of variance (ANOVA) with post-multiple comparison test (Dunnett) was performed (Supplemental Table S3).

Two different extraction volumes were compared (100 µL and 200 µL). Following extraction, different volumes for online-SPE were compared (PUFA: 10/20 µL, eicosanoids: 50/100 µL).

Reproducibility was assessed using pooled EV samples, which were aliquoted and stored at −80 °C. Extraction was performed according to the optimized protocol. Within-run variability was determined by measuring the pooled EV sample three times in one run. Between-run variability was calculated by measuring the extracted samples from the same EV pool on three consecutive working days. Coefficients of variation were calculated as the ratio of the standard deviation to the mean.

Sample stability was investigated by comparing analyte intensities after one or two FT cycles. Routinely, EV preparations are stored at −80 °C immediately after EV isolation. The sample preparation protocol usually involves an additional freezing step after lipid extraction. We modified the protocol by omitting this additional freezing step. Instead, after lipid extraction we immediately injected samples for LC-MS/MS analysis. The analyte intensities of both approaches were compared.

### 2.4. Data Analysis

Statistical analyses were performed using Microsoft Excel Office 2019 software (MS Office, Microsoft, Redmond, WA, USA) and GraphPad Prism 8 software (GraphPad Software, San Diego, CA, USA). The comparisons between multiple groups were calculated via one-way ANOVA with Dunnett follow-up multiple comparison test. For comparison of two groups, unpaired Student’s *t*-test was applied.

## 3. Results

### 3.1. Characterization of Isolated Extracellular Vesicles

**Figure 1:**
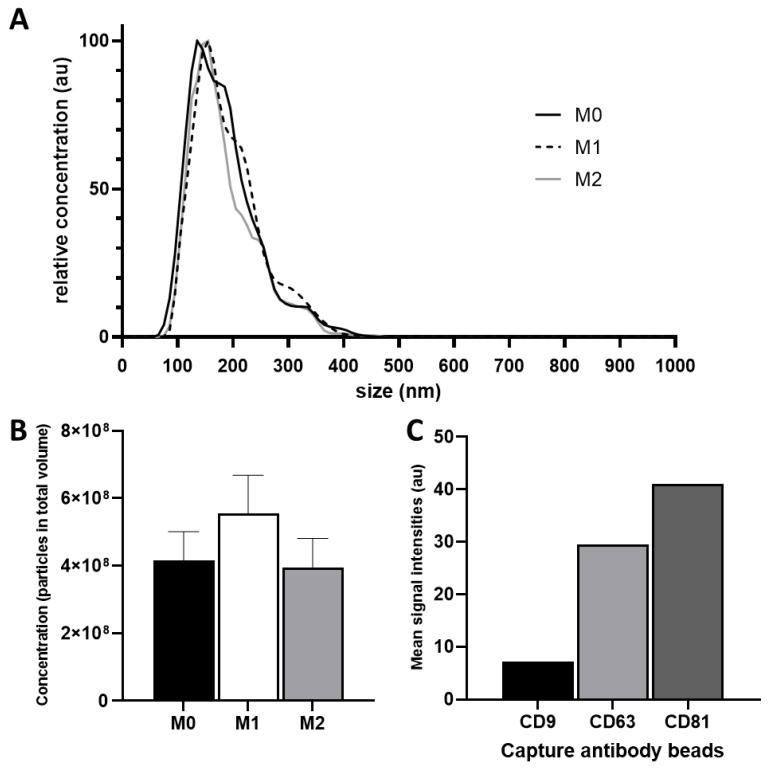
(**A**) Relative size distribution analysis of EV preparations. Three EV samples derived of differently polarized macrophages were isolated and prepared individually via TFF and assessed using NanoSight LM14 analyzer; (**B**) Mean particle concentrations of M0, M1 and M2; (**C**) Flow cytometry analysis of tetraspanin markers CD9, CD63, and CD81 on isolated EVs.

Isolated particles from M0, M1, and M2 macrophages showed uniform relative size distributions in all isolations. Particle sizes ranged from 35 nm to 165 nm, and the concentration maxima of the individual isolations lied between 135 nm and 155 nm (Figure 1A).

Total particle numbers in all isolations ranged between 3.99 × 10^10^ and 5.6 × 10^10^ particles. The highest quantity was measured in M1-derived EVs (5.6 × 10^10^ particles) and the lowest in M2-derived EVs (3.99 × 10^10^ particles). The mean particle concentration of M0, M1, and M2 EVs were 4.4 × 10^7^ particles/mL (M0), 5.8 × 10^7^ particles/mL (M1), and 4.2 × 10^7^ particles/mL (M2), respectively (Figure 1B).

Expressions of the previously-described EV surface markers CD9, CD63, and CD81 were investigated via flow cytometry. These markers were found in all three EV preparations (Figure 1C).

### 3.2. Development of a Sample Preparation Protocol for the LC-MS/MS Analysis of PUFAs and Eicosanoids for THP-1 Macrophage-Derived EVs

**Figure 2:**
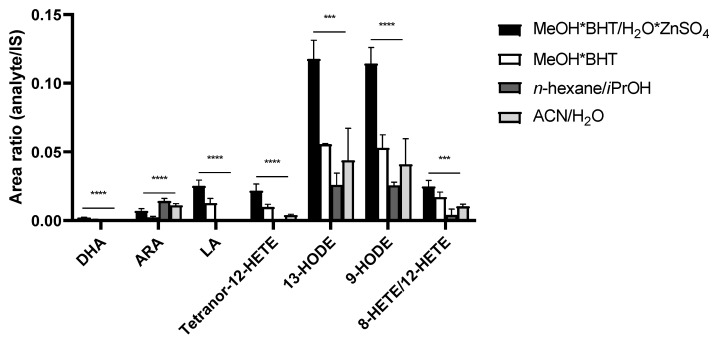
Comparison of precipitation solutions using pooled EV samples. Significant higher signals, expressed as peak area ratios of analyte to IS, for MeOH*BHT/H_2_O*ZnSO_4_ (80:20 *v*/*v*). Measurements performed as triplicates. Statistics: one-way ANOVA with follow-up multiple comparison test (Dunnett); *p* ≤ 0.001 (***), *p* ≤ 0.0001 (****). ACN—acetonitrile; ARA—arachidonic acid; BHT—butylated hydroxytoluene; DHA—docosahexaenoic acid; HETE—hydroxyeicosatetraenoic acid; HODE—hydroxyoctadecadienoic acid; iPrOH—propan-2-ol; IS—internal standard; LA—linoleic acid; MeOH—methanol.

The comparison of different precipitation reagents for lipid extraction is shown in Figure 2. The PUFAs DHA and LA were not detected using n-hexane/iPrOH (60:40 *v*/*v*) and acetonitrile/H_2_O (80:20 *v*/*v*), in contrast to ARA, which showed higher peak area ratios compared to MeOH*BHT/H_2_O*ZnSO_4_ (80:20 *v*/*v*) and MeOH*BHT. All detected eicosanoids were significantly increased using MeOH*BHT/H_2_O*ZnSO_4_ (80:20 *v*/*v*) for sample preparation. Accordingly, we continued using MeOH*BHT/H_2_O*ZnSO_4_ (80:20 *v*/*v*) for future EV sample preparation and optimization.

**Figure 3:**
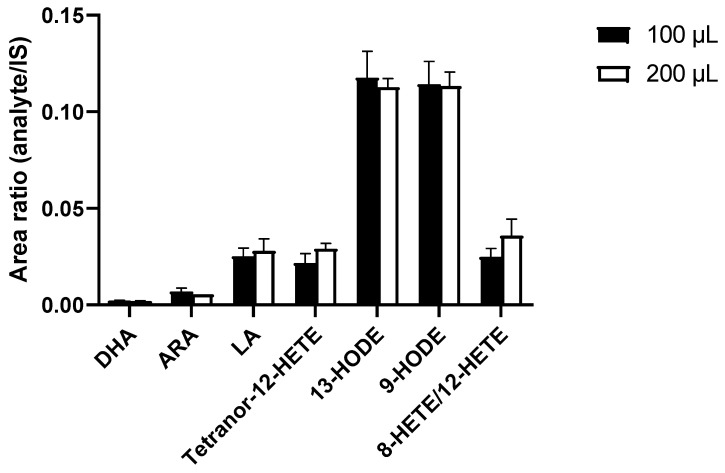
Increased sample volume for protein precipitation using MeOH*BHT/H_2_O*ZnSO_4_ (80:20 *v*/*v*) did not show any significant improvements in signal intensities, expressed as peak area ratios of analyte to IS after correction for dilution effects. Measurements performed as triplicates. Statistics: unpaired Student’s *t*-test, no significant differences. ARA—arachidonic acid; BHT—butylated hydroxytoluene; DHA—docosahexaenoic acid; HETE—hydroxyeicosatetraenoic acid; HODE—hydroxyoctadecadienoic acid; IS—internal standard; LA—linoleic acid; MeOH—methanol.

Different sample volumes for protein precipitation were compared, with the goal of improving PUFA and eicosanoid detection in EVs. Doubled sample volumes led to increased signal intensities and to no significant differences of analyte intensities in relation to IS intensities (Figure 3). Subsequently, the smaller sample volume was used in order to reduce consumption of precious samples.

We doubled the volumes injected onto the online-SPE and compared the resulting analyte peak areas to the analyte peak areas of the previously-used volumes [36]. The ratios (doubled volume/previous volume) were as follows: ARA 2.45, DHA 1.96, LA 1.93, Tetranor-12-HETE 1.74, 13-HODE 1.57, 9-HODE 1.51 and 8-HETE/12-HETE 1.43; the ratios of the internal standards were ARA-d8 2.08, DHA-d5 2.02, 5-HODE-d4 1.68, 5-HETE-d8 1.38.

**Figure 4:**
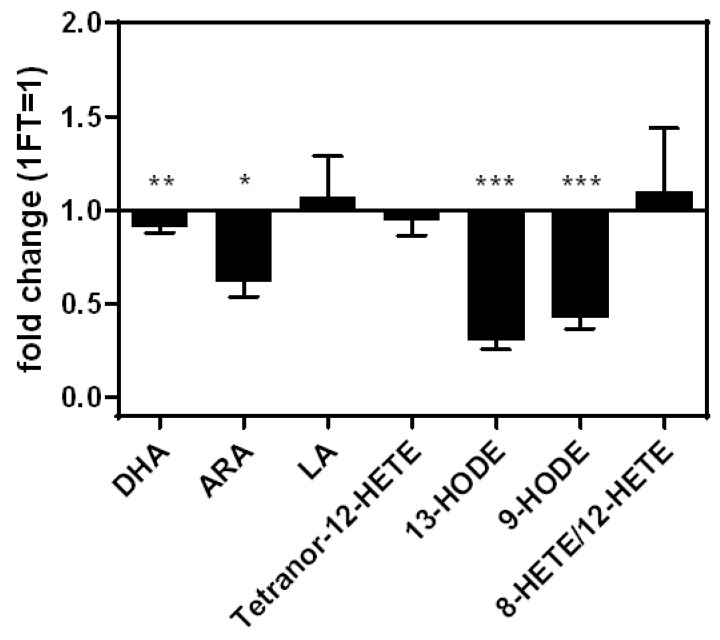
Fold changes of analyte concentrations comparing two freeze–thaw cycles relative to one freeze–thaw cycle. Measurements performed as triplicates. Statistics: unpaired Student’s *t*-test, *p* ≤ 0.05 (*), *p* ≤ 0.01 (**), *p* ≤ 0.001 (***). ARA—arachidonic acid; BHT—butylated hydroxytoluene; DHA—docosahexaenoic acid; FT—freeze-thaw cycle; HETE—hydroxyeicosatetraenoic acid; HODE—hydroxyoctadecadienoic acid; LA—linoleic acid; MeOH—methanol.

Two freeze–thaw (FT) cycles led to a significant reduction of the intensities of the PUFAs DHA (−16%) and ARA (−41%), compared to one FT cycle. The intensities of the eicosanoids 9-HODE (−59%) and 13-HODE (−71%) were significantly reduced as well. LA, Tetranor-12-HETE, and 8-HETE/12-HETE showed no significant changes in intensity (Figure 4).

**Figure 5:**
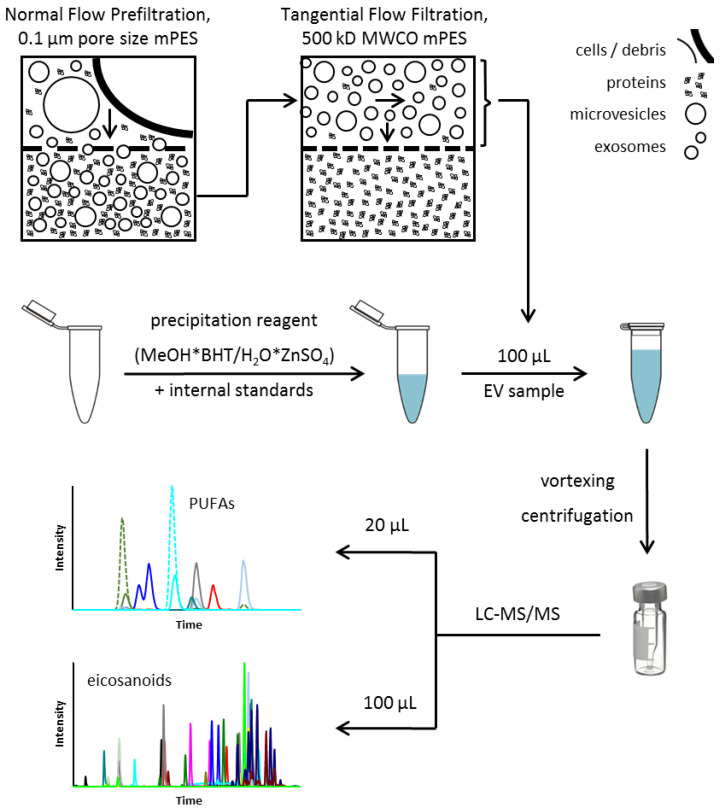
Final protocol for EV isolation and sample preparation for LC-MS/MS analysis of PUFAs and eicosanoids (adapted after [34]). BHT—butylated hydroxytoluene; EV—extracellular vesicles; LC-MS/MS—liquid chromatography—tandem mass spectrometry; MeOH—methanol; mPES—modified polyethersulfone; MWCO—Molecular Weight Cutoff; PUFA—polyunsaturated fatty acid.

After verification and optimization, the final sample preparation protocol from EV isolation to the analysis of bioactive lipid metabolites such as PUFAs and eicosanoids in EVs is illustrated in Figure 5.

Reproducibility of the optimized LC-MS/MS protocol for lipid analysis in EVs revealed within-run variability between 2.1% and 10.0%, as well as between-run variability of 5.2% to 13.3% for PUFAs and eicosanoids (Table 1).

### 3.3. PUFAs and Eicosanoids in M0, M1, and M2 Macrophage-Derived EVs

**Figure 6:**
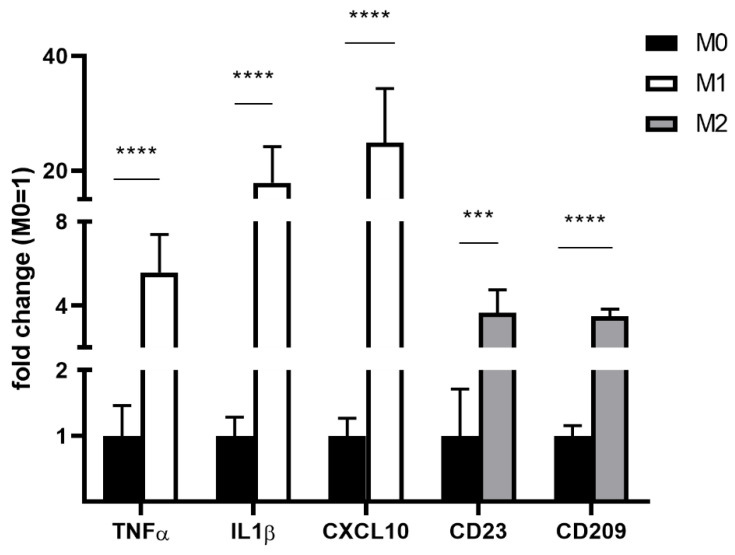
Expression of M1/M2 markers in differently polarized THP-1 macrophages as determined via RT-qPCR. Gene expression was expressed relative to beta-actin housekeeping gene expression and to resting M0 macrophages. Measurements were performed from *n* = 3 and as triplicates. Statistics: unpaired Student’s *t*-test; *p* ≤ 0.001 (***), *p* ≤ 0.0001 (****). CD—cluster of differentiation; CXCL—C-X-C motif chemokine ligand; IL1β—interleukin 1 beta; RT-qPCR—quantitative real-time polymerase chain reaction; THP-1—distinct human monocytic cell line; TNFα—tumor necrosis factor alpha.

Expression profiles of M1- and M2-polarized macrophages showed upregulation of typical markers, as previously described [30,31,37] (Figure 6).

Out of seven PUFAs and 94 eicosanoids detectable by our method (see Supplemental Figure S1), we repeatedly identified three PUFAs and eight eicosanoids in our EV preparations.

**Figure 7:**
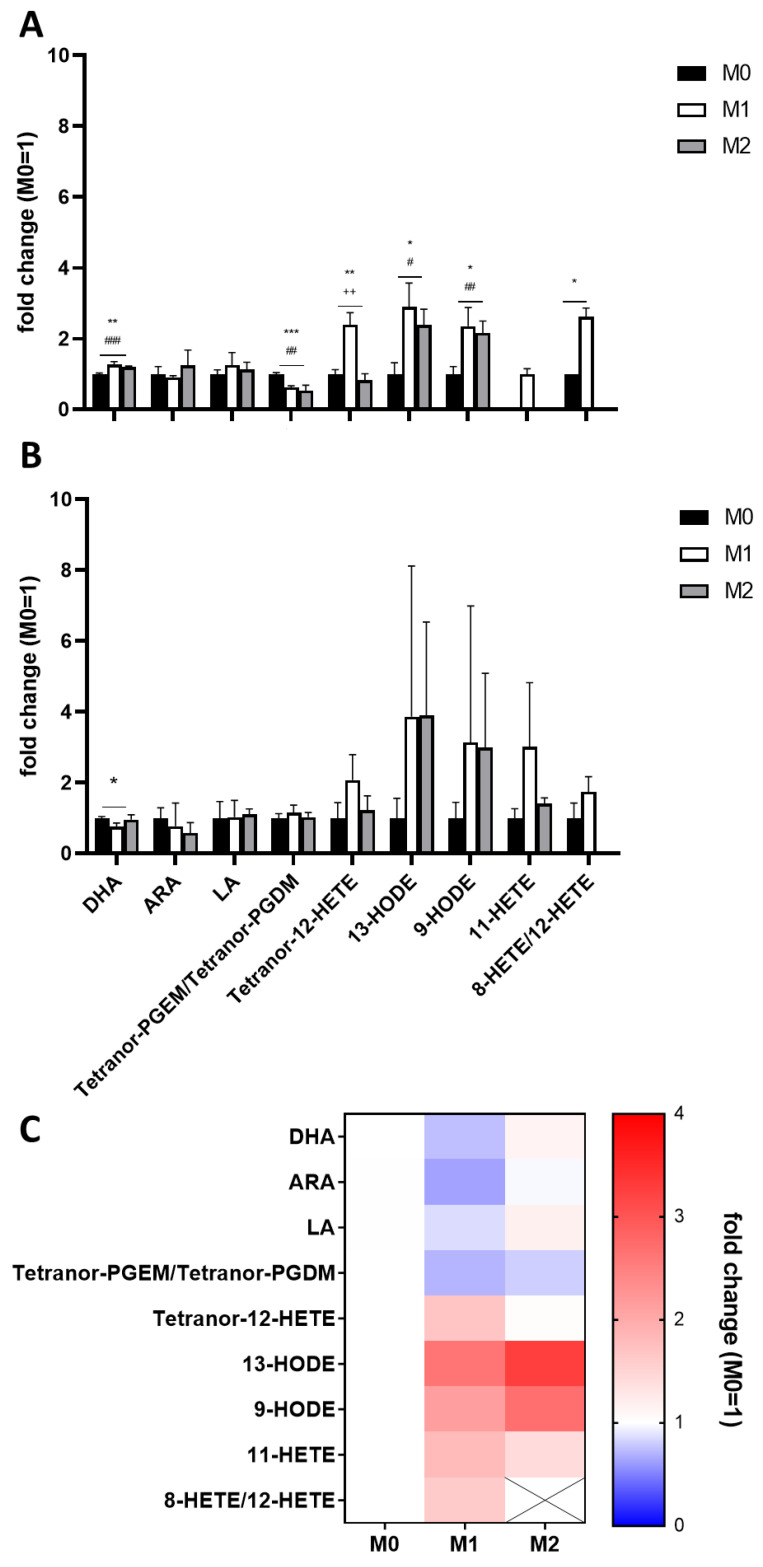
LC-MS/MS analysis of two independent EV isolations (**A**,**B**) of differently polarized THP-1 macrophages. Measurements were performed as triplicates and results are expressed as fold changes relative to M0. (**C**) Heatmap of the mean fold changes calculated from both EV isolations. Statistics: unpaired Student’s *t*-test; M0/M1 (*); M0/M2 (#), M1/M2 (+), *p* ≤ 0.05 (*/#), *p* ≤ 0.01 (**/##/++), *p* ≤ 0.001 (***/###). ARA—arachidonic acid; DHA—docosahexaenoic acid; HETE—hydroxyeicosatetraenoic acid; HODE—hydroxyoctadecadienoic acid; LA—linoleic acid; PGDM—prostaglandin D2 metabolite; PGEM—prostaglandin E2 metabolite.

In a first analysis, EVs derived from M1 macrophages showed significantly higher levels of Tetranor-12-HETE, 13-HODE, 9-HODE, 11-HETE, and 8-HETE/12-HETE and significant lower levels of Tetranor-PGEM/Tetranor-PGDM compared to EVs derived from resting (M0) macrophages (Figure 7A). EVs derived from M2 macrophages showed significantly higher levels of 13-HODE and 9-HODE and significantly lower levels of Tetranor-PGEM/Tetranor-PGDM compared to EVs derived from resting (M0) macrophages; 11-HETE and 8-HETE/12-HETE were not detectable in M2-derived macrophages (Figure 7A).

The duplicate analysis of a second independent differentiation and polarization experiment showed similar results, albeit no significant differences due to higher variations within the analyses (Figure 7B).

**Figure 8:**
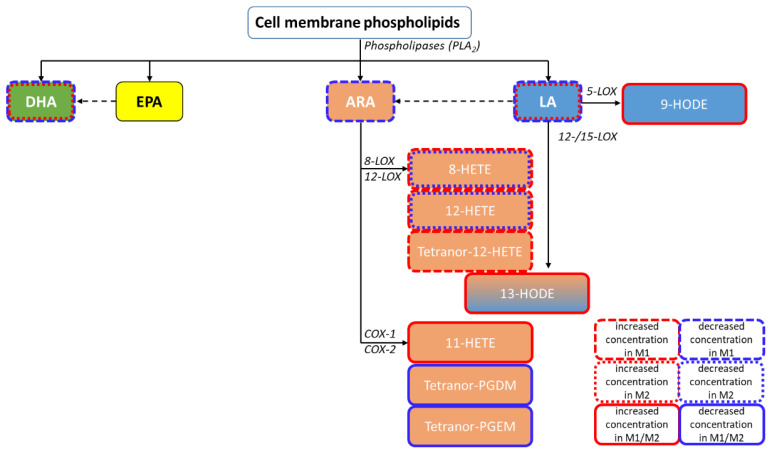
Pathway classification of the quantified PUFA metabolites within the performed independent EV isolations, including the most relevant enzyme classes. Increased concentrations compared to M0 are framed in red and decreased concentrations in blue. ARA—arachidonic acid; COX—cyclooxygenase; DHA—docosahexaenoic acid; EPA—eicosapentaenoic acid; HETE—hydroxyeicosatetraenoic acid; HODE—hydroxyoctadecadienoic acid; LA—linoleic acid; LOX—lipoxygenase; PLA2—phospholipase A2.

Mean concentrations of both analyses presented as a heatmap (Figure 7C) show increased levels of LA-derived eicosanoids in both M1- and M2-derived EVs compared to EVs derived of unpolarised macrophages. Furthermore, M1-derived EVs showed decreased levels of ARA but increased levels of ARA-derived 12-LOX and 15-LOX products (HETEs) (Figure 8). Detected COX products were decreased in both M1- and M2-derived macrophages; these findings are summarized in Figure 8.

## 4. Discussion

EVs play an important role in inflammation by enabling intercellular transport of proteins, nucleic acids, and bioactive lipid mediators. Thanks to advances in the field of lipidomic method development [9], bioactive lipids such as phospholipids, steroids, and glycerolipids have been described in EVs [26,29]. Recent studies have identified PUFAs and their eicosanoid metabolites in EVs [15,18,38]. While many of the underlying methodologies are untargeted, and thus give limited quantitative information for comparative analyses [17,27,28,39,40], other approaches allow quantitative detection of EV phospholipids, sphingolipids, glycerolipids, steroids, and fatty acids [26,29]. Few methods, however, have been described for quantitative assessment of EV PUFAs and eicosanoids [15,18,38]. Most of these methodologies only cover a limited analyte spectrum within these lipid species, and even involve (pre-)analytical sample modifications, e.g., incubation with ARA/Leukotriene A4 (LTA 4) or sample hydrolysis during the lipid extraction process [15]. Due to the lack of adequate quantitative methodologies, the contribution of eicosanoids to EV functions, as in inflammation, remains largely unknown.

When assessing EV lipid profiles, the EV isolation method has a large impact on the resulting lipid profiles. Most EV lipidomics methods described in the literature rely on UC-based EV isolation methods (see Supplemental Table S1), which are known to co-isolate particle aggregates such as free proteins [20,34]. As EVs isolated by UC reportedly have been described to contain enzymes of the PUFA metabolism that catalyze eicosanoid formation [15], it is not clear whether these enzymes are actually incorporated into EVs or whether their presence in EV isolations is a consequence of co-isolation of free proteins during UC-based EV isolation [20]. In order to prevent this issue, we isolated EVs by sequential filtration as previously described, including TFF [34,35]. By diafiltrating six times during TFF, we largely depleted free proteins smaller than the pore size of the hollow fiber filter used during TFF (500 kDa), and therefore assume depletion of free enzymes of PUFA metabolism (COX and LOX: 60 to 70 kDa) [41,42,43].

Another influencing factor of the EV isolation method is the respective time needed for EV isolation. As PUFAs are prone to in vitro auto-oxidation, a gentle and rapid EV isolation method should be used rather than lengthy ultracentrifugation-based methods. We adapted an established LC-MS/MS method for the analysis of PUFAs and eicosanoids which had been previously developed by our group for analysis of human plasma and tissue [21,36]. The method is capable of detecting seven PUFAs and 94 eicosanoids in human plasma.

The comparison of protein precipitation reagents such as MeOH*BHT/H_2_O*ZnSO_4_ (80:20 *v*/*v*), MeOH*BHT, n-hexane/iPrOH (60:40 *v*/*v*), and acetonitrile/H_2_O (80:20 *v*/*v*) confirmed that the precipitation solvent for plasma [36], MeOH*BHT/H_2_O*ZnSO_4_ (80:20 *v*/*v*), is suitable for EV sample pretreatment. The short sample preparation step of isolated EVs using protein precipitation combined with the addition of BHT as an antioxidant and ZnSO_4_ for yielding a fine precipitate allowed reduction of possible pre-analytical modifications through oxidation. In order to increase the number of detected analytes, we doubled the sample volume used for EV extraction; however, we were not able to identify additional analytes. Due to the limited sample availability of EVs, we continued method optimization with a 100 µL sample volume. Additionally, we increased the injection volume based on the previously-published plasma protocol [36]: By doubling the volume injected onto the online-SPE we observed doubled intensities of PUFAs and corresponding IS, as expected. The intensities of eicosanoids and corresponding IS, however, were much lower, with ratios between 1.74 and 1.38. We attribute these findings to lower analytical recovery of eicosanoids, which has been described previously [21,44]. We believe that varying analytical recoveries impede comparability of the results of different quantitative lipidomic methods. Within-run and between-run variability ranged between 2.1–13.3%, demonstrating good reproducibility and robustness of our adapted sample preparation protocol and the modified LC-MS/MS method.

While EVs have long been regarded as relatively stable during FT cycles, recent reports have shown reductions in particle and nucleic acid concentrations [24,45,46]. Accordingly, we show that varying pre-analytical conditions such as additional FT cycles have a large impact on EV lipid profiles. This is in keeping with previous works that described HETEs and HODEs as pre-analytically sensitive and unstable metabolites in blood [22,47]. The severe in vitro changes in EV PUFA and eicosanoid levels we identified during FT cycles show the importance of pre-analytics in EV lipid analysis and emphasize the need for further method standardization in the field. According to these and previous findings [21,22], we recommend immediate storage of isolated EV samples, omitting any further FT cycles.

A wide range of cell types contain 12-LOX and 15-LOX, including dendritic cells and macrophages [48,49,50], which represent one pathway in ARA metabolism. Their HETE products have pro-inflammatory properties such as induction of pro-inflammatory and pro-apoptotic cytokine expression, tumour progression, and chemotaxis [48,51,52,53,54]. ARA metabolism is known to be highly active in inflammation, especially in pro-inflammatory macrophages. Upregulation of LOX enzymes may then lead to increased HETE concentrations, which could explain our findings [7,49,50,55]. Compared to M0 EVs, we found decreased levels of the ARA-derived COX-1/2-products Tetranor-PGDM and Tetranor-PGEM in M1 EVs. These eicosanoids are metabolites of the pro-inflammatory mediators PGD2 and PGE2 [56,57], prostaglandins which are key players in inflammation and tumour progression [56,58,59]. However, both PGD2 and PGE2 are relatively unstable and susceptible to dehydration, impeding direct quantification in vitro [56,60,61]. After undergoing several metabolic steps involving enzymes such as prostaglandin dehydrogenase (PGDH), these prostaglandins are degraded into Tetranor-PGEM and Tetranor-PGDM. However, this degradation can happen in vitro as well due to reactive oxygen species (ROS) [62,63]. As none of the precursor molecules of Tetranor-PGEM and Tetranor-PGDM are detectable in any EV preparations, we assume that this finding is a pre-analytical issue. In M2-derived macrophages, ARA-derived eicosanoids were either detected in decreased concentrations or not detected at all, suggesting a significant suppression of the ARA pathway in M2 macrophages.

We could further see increased levels of the LA metabolites 9-HODE and 13-HODE in M1 EVs compared to M0 EVs. These eicosanoids are products of the 15-LOX enzyme pathway and are known to have pro-inflammatory and atherogenic effects [50,64] due to induction of increased Interleukin 1-beta (IL1b) expression in monocytes. An upregulation of these pro-inflammatory molecules during M1 polarization is a finding that was expected in our work and validates our approach. Interestingly, M2 macrophage-derived EVs showed similarly elevated levels of 13-HODE and 9-HODE compared to EVs derived from M0 macrophages. As these eicosanoids are associated with pro-inflammatory effects, this finding seems contradictory at first. However, this finding is explainable by LOX pathway activation by IL4 [49,65,66,67,68], which we used to polarize M2 macrophages. In the future, other M1/M2 macrophage differentiation approaches as well as the analysis of primary M1/M2 macrophages will allow confirmation of these findings.

M1 EVs showed no significant difference in DHA levels. Furthermore, no metabolites of DHA were detected. This is not surprising, as DHA and its metabolites are mostly associated with resolution of inflammation and therefore not expected to be upregulated in M1 macrophages.

Accordingly, M2 macrophage-derived EVs had slightly higher levels of DHA compared to M0 or M1 EVs. This is in keeping with previous findings that showed increased DHA concentrations in M2-polarized macrophages [49].

While the LC-MS/MS method shows reliable reproducibility and low between-run variation of the same samples, we observed high variations in individual fatty acid metabolites within biological replicates. Changes of key metabolites such as HODEs or HETEs, however, were reproducible. Low reproducibility is a previously-described issue when analysing PUFAs and their metabolites in plasma or EVs [15], and is usually explained by the challenging pre-analytics of PUFA metabolites [22]. Accordingly, a higher number of independent experiments is required in order to confirm up- or downregulation of individual analytes.

## 5. Conclusions

We present the most extensive quantitative LC-MS/MS based analytical procedure for the detection of PUFAs and eicosanoids in EVs to date. Fast and simple sample preparation in our work reduces pre-analytic in vitro modifications of lipid profiles. The protocol enables robust and reproducible EV lipid quantification and allows distinction of lipid profiles in EVs derived from differently polarized macrophages. However, pre-analytics remains a major issue, impeding reproducibility of biological replicates and comparability of the results of different methods. Therefore, further standardization is necessary, particularly of EV isolation processes.

## Figures and Tables

**Table 1:** Within-run (*n* = 3) and between-run (*n* = 3) variability of PUFAs and eicosanoids in an extracted EV pool sample.

Analyte		Within-Run (*n* = 3)		Between-Run (*n* = 3)	
		Mean ± SD	CV [%]	Mean ± SD	CV [%]
**DHA**	ng/mL	11.6 ± 0.2	2.1	11.1 ± 0.6	5.2
**ARA**		31.4 ± 1.5	4.7	31.7 ± 3.6	11.4
**LA**		61.7 ± 5.0	8.1	62.7 ± 4.9	7.9
**Tetranor-12-HETE**	pg/mL	11.1 ± 0.2	2.1	10.1 ± 1.2	11.6
**13-HODE**		14.7 ± 1.5	10.0	16.5 ± 2.2	13.3
**9-HODE**		15.1 ± 0.8	5.4	15.2 ± 1.4	8.9
**8-HETE/12-HETE**		10.0 ± 0.5	5.3	10.3 ± 1.1	10.5

ARA—arachidonic acid; CV—coefficient of variation; DHA—docosahexaenoic acid; EV—extracellular vesicle; HETE—hydroxyeicosatetraenoic acid; HODE—hydroxyoctadecadienoic acid; LA—linoleic acid; PUFA—polyunsaturated fatty acid; SD—standard deviation.

## Data Availability

Collected data are provided in the Supplementary Materials section.

## Supplementary Materials

The following supporting information can be downloaded at https://www.mdpi.com/article/10.3390/nu14071319/s1. Figure S1: Simplified PUFA metabolism with the most relevant enzyme classes; Figure S2: LC-MS/MS chromatogram of a standard mixture of (A) PUFAs, 1 µg/mL, (B) eicosanoids, 1 ng/mL; Table S1: Selection of mass spectrometric methods for lipid analysis of EVs; Table S2: Primer list for RT-qPCR targeting M1/M2 marker expression; Table S3: Comparison of precipitation solutions. Statistics: one-way ANOVA with follow-up multiple comparison test (Dunnett).
