# Supplementary material for: Targeted Lipidomics for Characterization of PUFAs and Eicosanoids in Extracellular Vesicles
Source: Nutrients. 2022 Mar 22;14(7):1319. doi: 10.3390/nu14071319 (PMC9000901; doi:10.3390/nu14071319)
Supplement: Supplementary file 1 [file nutrients-14-01319-s001.zip › nutrients-1632846-supplementary.pdf]

nutrients

Supplementary Materials

Targeted Lipidomics for Characterization of PUFAs and Eicosanoids in Extracellular Vesicles

Madlen Reinicke †, Saikal Shamkeeva †, Max Hell, Berend Isermann, Uta Ceglarek and Mitja L. Heinemann *

Institute of Laboratory Medicine, Clinical Chemistry and Molecular Diagnostics, Leipzig University Hospital, Paul-List-Str. 13-15, 04103 Leipzig, Germany; madlen.reinicke@medizin.uni-leipzig.de (M.R.); saikal.shamkeeva@medizin.uni-leipzig.de (S.S.); mhell97@gmx.de (M.H.); berend.isermann@medizin.uni-leipzig.de (B.I.); uta.ceglarek@medizin.uni-leipzig.de (U.C.)

* Correspondence: mitja.heinemann@medizin.uni-leipzig.de (M.L.H.)

† These authors contributed equally to this work.

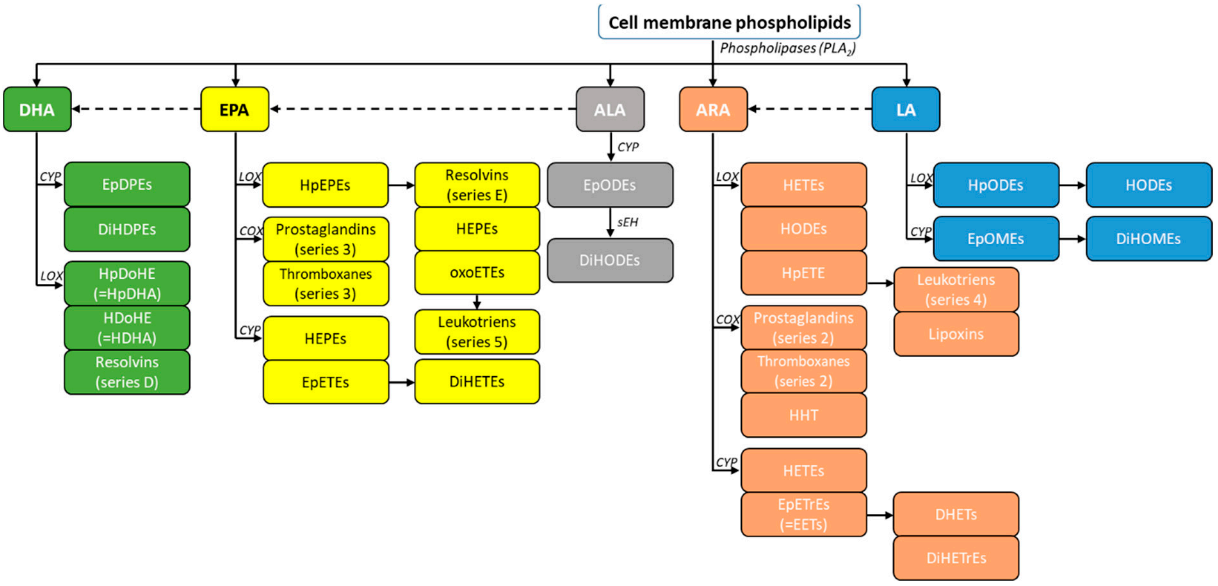

Figure S1. Simplified PUFA metabolism with the most relevant enzyme classes. ALA - alpha linolenic acid; ARA - arachidonic acid; COX - cyclooxygenase; CYP - cytochrome P450; DHA - docosahexaenoic acid; DHET - dihydroxyeicosatrienoic acid; DiHETrE - dihydroxyeicosatrienoic acid; DiHDPE - dihydroxydocosapentaeinoic acid; DiHETE - dihydroxyeicosatetranoic acid; DiHODE - dihydroxyoctadecadienoic acid; DiHOME - dihydroxyoctadecamonoenoic acid; EPA - eicosapentaenoic acid; EpDPE - epoxydocosapentaeinoic acid; EpETE - epoxyeicosatetraenoic acid; EpETrE (=EET) - epoxyeicosatrienoic acid; EpODE - epoxyoctadecadienoic acid; EpOME - epoxyoctadecamonoenoic acid; HDoHE (=HDHA) - hydroxydocosahexaenoic acid; HEPE - hydroxyeicosapentaenoic acid; HETE - hydroxyeicosatetraenoic acid; HHT - hydroxyheptadecatrienoic acid; HODE - hydroxyoctadecadienoic acid; HpDoHE (=HpDHA) - hydroperoxydocosahexaenoic acid; HpEPE - hydroperoxyeicosapentaenoic acid; HpETE - hydroperoxyeicosatetraenoic acid; HpODE - hydroperoxyoctadecadienoic acid; LA - linoleic acid; LOX - lipoxygenase; oxoETE - oxoeicosatetraenoic acid; PLA2 - phospholipase A2; PUFA - polyunsaturated fatty acid; sEH - soluble epoxide hydrolase.

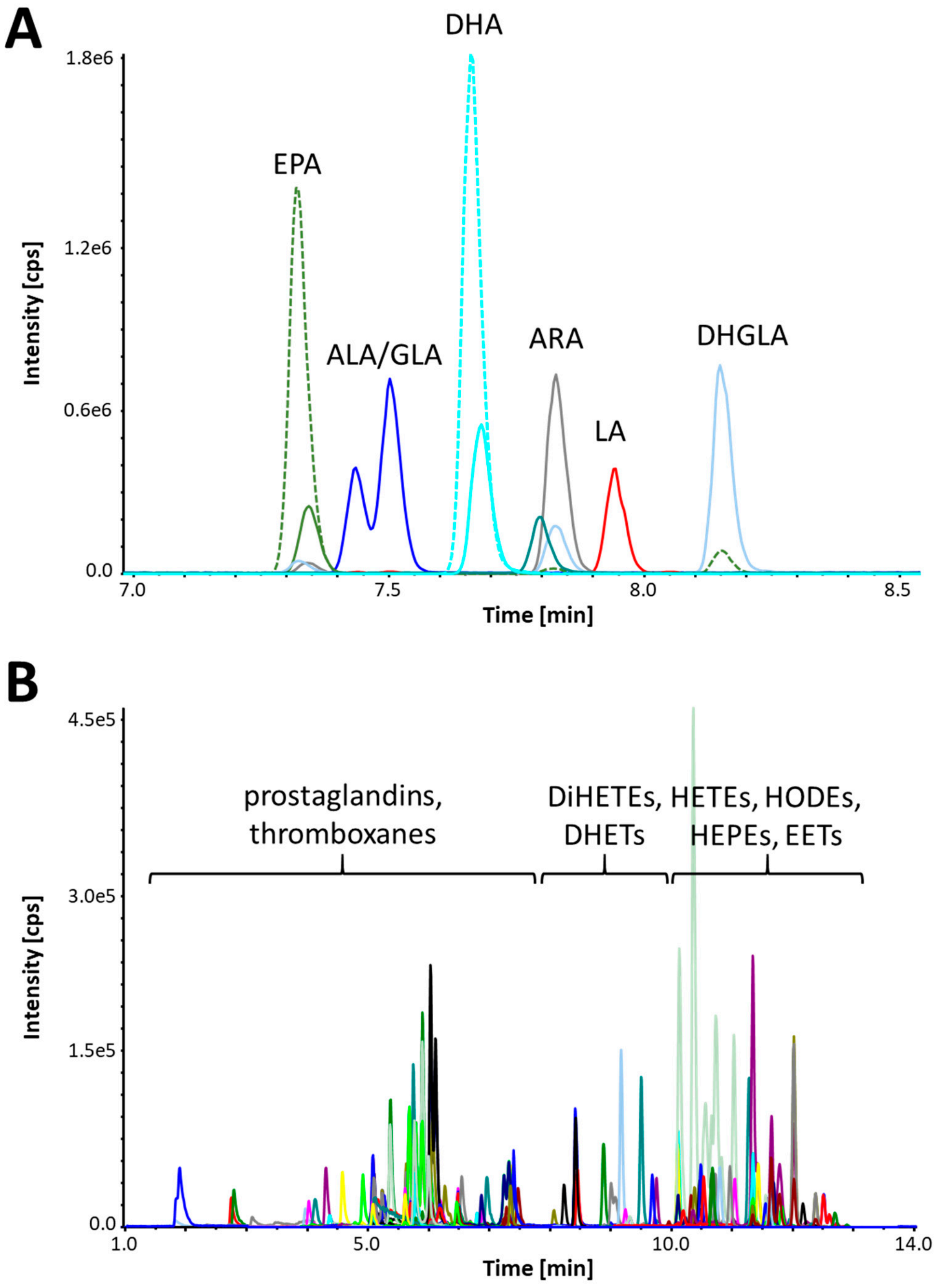

Figure S2. LC-MS/MS chromatogram of a standard mixture of (A) PUFAs, 1 µg/mL, (B) eicosanoids, 1 ng/mL. ALA - alpha linolenic acid; ARA - arachidonic acid; DHA - docosahexaenoic acid; DHET - dihydroxyeicosatrienoic acid; DHGLA - dihomo-gamma-linolenic acid; DiHETE - dihydroxyeicosatetranoic acid; EET - epoxyeicosatrienoic acid; EPA - eicosapentaenoic acid GLA - gamma linolenic acid;HEPE - hydroxyeicosapentaenoic acid; HETE - hydroxyeicosatetraenoic acid; HODE - hydroxyoctadecadienoic acid; LA - linoleic acid; LC-MS/MS - liquid chromatography - tandem mass spectrometry; PUFA - polyunsaturated fatty acid.

Table S1. Selection of mass spectrometric methods for lipid analysis of EVs.

Author	EV Isolation method	Lipid extraction	Method for lipid analysis	Lipid analytes
Subra *et al.* 2010 (18)	ultracentrifugation	Bligh and Dyer (25) and ethyl acetate	GC-MS	differently saturated FA (C_{14}-C_{24}), PG series 2
Esser *et al.* 2010 (15)	ultracentrifugation / filtration	chloroform/MeOH (2:1) followed by hydrolysis	LC-MS/MS	LT serie 4, 5-HETE, 5-KETE, 15-HETE, 12-HETE, 15-KETE (samples were incubated with ARA, LTA_4)
Llorente *et al.* 2013 (26)	ultracentrifugation	chloroform/MeOH (2:1)	LC-MS/MS	HexCer, LacCer and SM lipid classes; PC lipid class
Duchez *et al.* 2015 (16)	ultracentrifugation	chloroform/MeOH (2:1)	LC-MS/MS	12-LOX (Enzyme), 12-HETE
Haraszti *et al.* 2016 (27)	ultracentrifugation	chloroform/MeOH (1:1)	MS/MS	untargeted lipidomics, including differently saturated FA (C_{12}-C_{22}),
Dang *et al.* 2017 (72)	ultracentrifugation / filtration	MeOH/DCM (2:0,9)	MS/MS	untargeted lipidomics, including differently saturated FA (C_{14}-C_{22})
Brzozowski *et al.* 2018 (29)	ultrafiltration	chloroform/MeOH (1:1)	LC-MS/MS	glycerolipids, glycerophospholipids, sphingolipids, fatty acids and sterol lipids
Hough *et al.* 2018 (17)	ultracentrifugation	chloroform/MeOH (1:2)	MS/MS	untargeted lipidomics, including sphingomyelins, glycerophospholipids and ceramides
Chen *et al.* 2019 (40)	ultracentrifugation	MeOH/DCM (1:1) with 5 mM ammonium acetate	MS/MS	untargeted lipidomics, including saturated and unsaturated species (e.g. Cer, LPC, PC)
Lacy *et al.* 2019 (73)	ultracentrifugation	directly from precipitate	LC-MS/MS	PG series 2
Nishida-Aoki *et al.* 2020 (28)	ultracentrifugation / filtration	MeOH/chloroform/water (10:5:3)	MS/MS	untargeted lipidomics, including FA, cholesterol, CE and TG
Surmiak *et al.* 2020 (38)	ultracentrifugation / filtration	tert-buthyl-ether/MeOH (80:20) after acidification	LC-MS/MS	LT series 4, 5-HETE, 12-HETE, 5-oxo-ETE, 13,14-dihydro-15-keto-PGE_2

ARA - arachidonic acid; Cer - ceramides; COX - cyclooxygenase; CE - cholesteryl ester; DCM - dichloromethane; DHA - docosahexaenoic acid; EV - extracellular vesicle; ETE - eicosatetraenoic acid; FA - fatty acid; GC - gas chromatography; HETE - hydroxyeicosatetraenoic acid; HexCer - hexosylceramide; KETE - ketoeico-

satetraenoic acid; LacCer - lactosylceramide; LOX - lipoxygenase; LT - leukotriene; LPC - lysophosphatidylcholine; LC-MS/MS - liquid chromatography - tandem mass spectrometry; LXA4 - Lipoxin A4, MeOH - methanol; PC - phosphatidylcholine; PG - prostaglandin; PGE2 - prostaglandin E2; PUFA - polyunsaturated fatty acid; SM - sphingomyelin; TG – triacylglycerol.

Table S2. Primer list for RT-qPCR targeting M1/M2 marker expression.

Number	Oligoname	Gene	Sequence	Target
**Pr1**	hACTB-F	ACTB	ATTGCCGACAGGATGCAGAA	THP-1 (M0)
**Pr2**	hACTB-R	ACTB	GCTGATCCACATCTGCTGGAA	THP-1 (M0)
**Pr3**	hTNF-a_F	TNF-alpha	CTTCTGCCTGCTGCACTTTG	THP-1 (M1)
**Pr4**	hTNF-a_R	TNF-alpha	GGCCAGAGGGCTGATTAGAGA	THP-1 (M1)
**Pr5**	hIL-1B_F	Interleukin-1-Beta	CAGTGGCAATGAGGATGACTTG	THP-1 (M1)
**Pr6**	hIL-1B_R	Interleukin-1-Beta	AGTGGTGGTCGGAGATTCGT	THP-1 (M1)
**Pr7**	hCXCL10_F	C-X-C motif chemokine 10	ATTCCTGCAAGCCAATTTTGTC	THP-1 (M1)
**Pr8**	hCXCL10_R	C-X-C motif chemokine 10	CATCTCTTCTCACCCTTCTTTTTCA	THP-1 (M1)
**Pr9**	hCD23_F	CD23	CCCGGAACGTCTCTCAAGTTT	THP-1 (M2)
**Pr10**	hCD23_R	CD23	TCAGCTCGAAGTTCCTCCAGTT	THP-1 (M2)
**Pr11**	hCD209_F	CD209 / DC-SIGN	TCAAGCAGTATTGGAACAGAGGA	THP-1 (M2)
**Pr12**	hCD209_R	CD209 / DC-SIGN	CAGGAGGCTGCGGACTTTTT	THP-1 (M2)

Table S3. Comparison of precipitation solutions. Statistics: one-way ANOVA with follow-up multiple comparison test (Dunnett).

p value	one-way ANOVA	multiple comparison test (Dunnett)		
		A vs. B	A vs. C	A vs. D
**DHA**	<0.0001	<0.0001	<0.0001	<0.0001
**ARA**	<0.0001	0.0124	0.001	0.0261
**LA**	<0.0001	0.0012	<0.0001	<0.0001
**Tetranor-12-HETE**	<0.0001	0.0016	<0.0001	0.0001
**13-HODE**	0.0003	0.0018	0.0001	0.0006
**9-HODE**	<0.0001	0.0007	<0.0001	0.0002
**8-HETE/12-HETE**	0.0006	0.0711	0.0003	0.0031

A: MeOH*BHT/H2O*ZnSO4 (80:20 v/v, 56 mg/L BHT, 17.8 g/L ZnSO4), B: MeOH*BHT (56 mg/L BHT), C: n-hexane/iPrOH (60:40 v/v), D: ACN/H2O (80:20 v/v)
